# Dental Tumors

**Published:** 1868-09

**Authors:** Am. Forget

**Affiliations:** Member of the Imperial Society of Surgeons.


### ARTICLE IV.

### *Dental Tumors.*

VARIETY, DIAGNOSIS AND TREATMENT.

By Am. Forget, Member of the Imperial Society of Surgeons.

(Translated from the French.)

The study of the tumors of the maxillary bone produced by the hypergenesis of the odontogenetic tissues, was all to be begun a dozen years ago. At that time, having been obliged to practice resection of the left half of the lower jaw, in a young man of twenty years of age, I ascertained that, that part of the maxilla, transformed into a large cavity, contained a tumor having the form and size of a large egg, of the consistance of ivory, with the surface sprinkled with little irregular points covered with a layer of enamel. Between this tumor, firmly imbedded in a bony cyst, and the walls of the latter, then was a thick grayish membrane, bathed in a semipurulent liquid.

Anatomical examination of the tumor left no doubt on my mind as to its nature and origin. Originating in excessive development of the dental elements, it was wholly composed of dentine in its centre and of a layer of bony tissue or cementum, at its base, especially on the periptery.

It was interesting to know under what condition this pathological production, without analogy in the records of science, had been developed. We learned that at the age of five years, our patient had felt the first attack of the disease. He experienced acute pain in the left side of the jaw. For some time intermittent, these pains became continuous, and so severe that at the age of seven, two small molars were extracted, although they were healthy, under the impression that they were interfering with the evolution of the permanent teeth. This operation relieved the pain, but soon afterwards, a rounded tumor, the size of a hazel nut was found on external face of the jaw, over the alveoli where the teeth had been extracted.

Stationary for eight years, the tumor then assumed more considerable proportions, one half of the jaw swelling and assuming the oval form which it retained. A year later, in 1854, intense inflammation seized upon the tumor, successive abscesses opened on the inside and outside of the mouth, at the base of the jaw, and then the young man came to consult me.

This clinical observation and the pathological product set me upon making histological researches, the result of which I embodied in a memoir crowned by the Academy of Science, of which the reporter of the committee on prizes, M. Velpeau, observed that it contained a new fact, viz: the discovery of tumors which may be developed in the thickness of the maxillary bones, the origin and nature of which had not heretofore been examined.

I demonstrated that these tumors must be considered as anomalies of the nutrition and development of the teeth, that they are a modification of the physiological type, and that they can exist in different stages of growth and of volume.

Limited and circumscribed to one point of the dental bony tissue, the tumor represents, so to say, the first step taken by nature outside of the regular province of normal evolution; it is a sort of commencement of a considerable development altogether unusual, like that which I have related at the beginning.

Besides, and I took care to say so, at whatever stage of development we observe it, whether with its natural type or in the most advanced form, this anomaly, is in all cases, only explained by a primitive normal condition of the dental follicles, since the dental tissues, destitute of any vascular element, cannot of themselves be the seat of any nutritive or pathological change. Hence, in order to account for the alterations of form and structure which they present, we are compelled to go back to their formation, for it is only through it that they belong to the organism and that they have under gone the influence of the laws of formation which govern it.

It is not however, masses of dentine only due to excess of development of one or several dental bulbs, which can show themselves upon the interior of the jaw; I have also observed, alongside of the normal tooth, the existence of other morbid products, having with them more or less intimate connection and capable also of entirely independent existence.

The tooth which presents this anatomical anomaly, brought about as I have determined by the microscope with the assistance of M. A. Robin, by the production of cementum in excess, generally preserves its physiological characters, and the alveolus which is its receptacle, is not sensibly modified in its form so long as the tumors, almost always confined to the roots of the teeth, have acquired little development; so that generally they are for a long time inoffensive, and the patients remain unconçious of their existence.

When however, they grow sufficiently to alter the conditions of vitality and the forms of the jaws, they require the intervention of the surgeon. M. Letenneur, of Nantes, submitted a case of this kind to the consideration of the Society of Surgery in 1858, under the title of *exostosis eburnea* and which M. Guyot brought up again at the meeting of the 18th of last March. This hitherto unpublished case will be read with interest.

*Dental tumor formed of bony tissue and cement, (odontome cementaire,) considerable development of the left side of the lower jaw, intra-maxillary abcess, operation, cure.*

The woman V., aged 34 years, entered the Hotel Dien at Nantes, February 8th, 1858, with a tumor of the lower jaw. This woman says that, about the age of 8 years, she experienced pains in the left side of the lower jaw; the removal of a large diseased molar produced but imperfect relief. From this time the left side of the jaw was larger than the right, and this swelling did not diminish after the extraction of the tooth. Matters remained in this condition for sixteen years, and save the swelling of the jaw and some trifling pains, every thing appeared to have returned to its natural condition.

After these sixteen years, new and severe pains supervened and recourse was had to emollients and leeches. The sharp pains again subsided, every thing returned to its habitual condition and the tumor did not sensibly increase. Towards the close of 1857, sixteen years after the occurrence of the first pains, the trouble became more serious. It was attributed to a carious tooth. This the patient had extracted, without, however any cessation of the pain, which, on the contrary greatly increased. The swelling became enormous, and several abscesses opened in the mouth and remained fistulous. For six months then was no new symptom, but the patient was constantly annoyed by the pus which ran into the mouth and by the size of the tumor. About this time, a country quack advised the application of a plaster, which was kept on for fifteen days. During this time the pain was intolerable, and when the diseased region was uncovered, it, was found that a deep wound had been produced which reached the bony tissue. From this time the pus escaped outwardly, but the tumor increased, especially in the inside of the mouth. At this period the patient came under my treatment.

I found, continues M. Letenneur, a considerable swelling of the lower jaw, the two layers of which appeared to be separated from that of the tumor. The tumor was not circumscribed and its limits could not be fixed. A stylet introduced into the fistula, reached the centre of the tumor and touched a movable wrinkled sequestrum, offering to percussion and pressure the character of a very compact mass.

An incision was made dividing the lower lip on the medium line, then following the base of the jaw to the masseter muscle. The flap was turned back, and the bony shell exposed by cutting pincers. Aided by the gouge and mallet, I removed the whole external wall and was able to extract a bony mass to which adhered a small molar. This bony mass resembled a mural calculus. The internal wall of the bony cyst was excised in like manner with the external wall, the flap was returned to its position and retained by suture.

Union took place by first intention and in fifteen days the patient left the hospital, with the old fistula in the lower part of the cheek, through which ran a little saliva.

In spite of my request that she would return I did not see her for six years. During all this time, the fistula remained open and poured out an abundance of saliva, the inconvenience of which she remedied by a little plug of lint. I induced the patient to submit to a second operation, which was entirely successful, and now scarcely any traces of the double operation are visible.

The examination of the tumor with a lens proved to M. Letenneur its great hardness and its ivory consistence. The microscope revealed its bony structure by establishing the presence of a number of osteoplasts. This tumor which is formed of cementum, was attached to the first small molar which adhered to it by the extremity of its root, as if to bury itself in the abnormal tissue. Still the whiteness of the root contrasted with the grayish tint of the neoplasm. Its greatest diameter, including the root of the tooth, is 3½ centimetres, the other diameters are at least 1 centimetre.

It seems desirable to place by the side of this observation a fact with which it has great analogy, the first which has been clinically studied in man; it occurs in my memoir on dental anomalies, where it has served me as a basis for the description of this variety of dental tumors.

*Case—Bony intra-maxillary tumor attached to an adjoining molar tooth. Simultaneous removal of this tooth and the tumor.*

A man aged 45 years, came from the country to Paris, to have a tumor removed from the interior of his mouth, which annoyed him and gave him severe pain. This tumor occupied the left side of the lower jaw and formed a decided protuberance both on the outside and inside of the jaw, but especially on the outside, producing a disagreeable distortion of the features. At the smaller extremity of the oval tumor, was a carious tooth the crown of which, imperfectly destroyed, was almost hidden by the protrusion of the gum raised by the morbid product within the aveolus. Before treating this tumor, my learned colleague, Mr. Maisonneuve, to whose politeness I owe the specimen, induced the patient to have his carious tooth extracted, thinking that thereby he would make an opening for the better exploration and more easy attack of the encysted product. This preliminary operation had the unexpected result of finishing the whole case, for the tooth and tumor came away at the the same time.

This tumor, which was larger than a pigeon's egg, was fastened to the root of a molar tooth by a very narrow pedicle. The examination of the specimen which Mr. Robin himself made at my request, showed that it was composed of cementum or bony tissue.

The similarity of origin, structure and position of these dental tumors, justify the opinion I have expressed of their histogeny and of the chemical characteristics which distinguish them from other encysted tumors of the maxillary bones.

All have the common tendency to clothe themselves with an enveloping membrane, which is nothing but the alveolo-dental periosteum, and which bears the same relation to them that it does towards the roots of the teeth themselves, in the regular conditions of their development. It is this membrane, at least in man, which according to certain anatomists, has no special organ for the production of cementum, which secretes the bony elements, constituting by their aggregation these cementum tumors, heretofore wrongly regarded as dental exostoses.

To consider them in this light is to fix upon them all the physiological theories which belong only to the immediate dependents on the bony system; it is to admit that they take their origin from the tooth itself, while in reality we have to do with a substance entirely distinct, the formation and growth of which are utter strangers to the organic operation to which the latter owes its development.

I have said that these bony neoplasms could exist wholly independent of the neighboring tooth. This absence of all original identity between it and the cementum tumors we are considering, is completely demonstrated by a pathological observation on a horse, which has furnished me with a specimen figured in the atlas appended to my work. The figure shows the neoplasms isolated on all sides, without any connexion with the carious tooth, against which it simply rested. This tooth, unusually developed, was thinnest toward the centre of the jaw, to make room for the morbid growth. The latter, irregular in shape, wrinkled, unequal on certain points of its surface, was as large as an egg. The progressive alteration of the bony cyst which enclosed it, the formation of an abcess in its interior, and its sudden opening by ulceration, was the accident to which it gave rise and which revealed its existence and exhibited its character.

It is very difficult, it must be acknowledged, to get a clear idea of this last, especially at the commencement of the evolution of the neoplasm, when the vague and ill-determined symptoms give no idea of the physiological changes which are going on. It is remarkable that the first symptoms occur at the time when the second dentition excites and keeps up in the jaws a fullness of blood and a more active vitality, evinced by neuralgic pains of extreme severity and unusual persistence.

When the symptoms cease to be transitory, when they are intensified and followed by a hard round tumor confined to the alveolus corresponding to the roots of growing teeth, there is a strong presumption that the tumor belongs to the class under consideration.

As for the time requisite for such a tumor to produce inflammation and suppuration of the bony cyst and the destruction of its walls, that is always very long. The case reported by Mr. Letenneur corresponds with my own observations, since it was twenty-six years before the neoplasm reached this last stage of its evolution.

If I insist upon what relates to the very obscure diagnosis of these tumors of cementum, it is because the practical point which I have attempted to deduce from my personal researches is precisely to bring out as strongly as possible the primordial characters which can put the surgeon upon the track of these anomalies of nutrition and physiological increase, and permit him to anticipate their pathological consequences.

If they already exist and result in more or less voluminous tumor of the jaw, the end which I then propose to myself and which I have endeavored to bring before the profession, is to open to diagnosis a way which, by rendering the tumor accessible, restricts the operation to the diseased points only, and enables us to preserve the continuity of the maxillary arch, the partial or total resection of which, excusable at a time when most tumors were badly known and confounded with one another, is no longer justifiable now that pathological anatomy has established distinctive characters which do not permit us any longer to misunderstand the perfect homology of these which belong to dental evolution

In this short treatise, we have discussed only the two varieties of dental tumors which I described in 1859, one formed by the overgrowth of dental tissue, the other by the abnormal production of bony tissue about the root of the teeth. There is a third variety formed by the dental bulb the hypertrophied elements of which consist of these fibrous bodies of the jaws the nature of which had been constantly misunderstood till our time. This variety has been studied in a memoir which I published in 1861, based upon the examination of a unique specimen, which my learned colleague Mr. Letenneuer, was kind enough to send me.

